# Causal relationship between asthma and chronic rhinosinusitis: A Mendelian randomization study

**DOI:** 10.1097/MD.0000000000045085

**Published:** 2025-10-10

**Authors:** Jumei Li, Derong Zhu, Zhonglin Mu

**Affiliations:** a Department of Otolaryngology-Head and Neck Surgery, The First Affiliated Hospital of Hainan Medical University, Haikou, Hainan, China.

**Keywords:** asthma, chronic rhinosinusitis, Mendelian randomization

## Abstract

Observational studies have shown an association between asthma and chronic rhinosinusitis (CRS), but the exact causal relationship has not been clarified. This study aimed to deeply explore the causal relationship between asthma and CRS by using a 2-sample bidirectional Mendelian randomization (MR) method. This study used a 2-sample MR method, utilizing genome-wide association study data on asthma from the UKB database with a sample of 56,167 asthma patients and 3,52,255 controls, and CRS genome-wide association study data from the FinnGen database with a total of 8524 CRS patients and 1,67,849 controls. We conducted causal analyses through the inverse-variance weighting method, MR-Egger method, weighted median method, and weighted mode method, applied Cochrane’s *Q* test for heterogeneity, and the MR-Egger intercept test for horizontal pleiotropy. At the same time, we implemented the MR-PRESSO and “leave-one-out” tests to verify the stability of the results. The analysis results showed a positive correlation between asthma and CRS, with an odds ratio of 1.320 (95% confidence interval = 1.206–1.444, *P* < .001). The results did not show heterogeneity or horizontal bias (*P > *.05), and the “leave-one-out” test also confirmed the reliability of this association. In the reverse MR analysis, no causal association was observed between CRS and asthma, with an odds ratio of 1.030 (95% confidence interval = 0.998–1.062, *P* = .065). Similarly, no horizontal bias or heterogeneity was found (*P > *.05), and the “leave-one-out” test also verified the reliability of this result. The results indicate that asthma may increase the risk of developing CRS, while CRS does not seem to increase the risk of developing asthma. This finding provides new insights into the complex causal relationship between the 2 and offers important evidence for the prevention and treatment of related diseases in the future.

## 1. Introduction

Asthma is a chronic lung disease characterized by bronchospasm, usually caused by anaphylaxis or anaphylaxis, resulting in symptoms such as dyspnea, wheezing, and coughing, which imposes a huge burden on patients and society worldwide.^[1]^ According to the global burden of disease study, there were an estimated 262 million cases of asthma worldwide in 2019 and an estimated 4,61,000 deaths from asthma.^[2]^ Chronic rhinosinusitis (CRS) usually refers to inflammation of the mucosa of the nasal cavity and paranasal sinuses lasting for more than 12 weeks. This condition may be accompanied by symptoms such as nasal congestion, facial pain or pressure, decreased sense of smell, and nasal reflux, and some patients may also develop nasal polyps.^[3]^ The global prevalence is 2% to 16%.^[4]^

In recent years, the causal relationship between asthma and CRS has been controversial.^[5]^ Studies indicate that approximately 20% to 33% of patients with CRS also have asthma, which is 4 times the prevalence of asthma in the general population.^[6]^ In addition, studies have shown a positive correlation between the severity of asthma and the evidence of chronic sinusitis found by imaging.^[7]^ These results highlight a potential link between the 2 diseases. However, current studies have been inconsistent. For example, some studies based on spirometry have found no significant correlation between CRS and asthma severity.^[8]^ In addition, studies of endoscopic scores in patients with chronic sinusitis have also shown no significant correlation between oral corticosteroids and chronic sinusitis in the past year.^[9]^ These findings suggest that the link between asthma and CRS may be influenced by a variety of factors, and the nature of this relationship still needs to be clarified by further research.

In recent years, Mendelian randomization (MR) studies have been increasingly recognized in the field of epidemiology and are often described as naturally occurring randomized trials.^[10]^ This method uses single nucleotide polymorphisms (SNPs) as an instrumental variables (IVs) to assess whether there is a correlation between exposure and outcome.^[11]^ In MR, the delivery of a single nucleotide is transmitted to offspring according to the principle of random allocation of alleles, similar to RCTs. This process effectively avoids confounding factors and reverse causal interference that may exist in observational studies.^[12]^ The bidirectional 2-sample MR method was used to investigate the potential causal relationship between asthma and CRS. The genome-wide association study (GWAS) data used in this study were derived from published studies, and all study participants had already obtained informed consent in their respective original studies, so no additional ethical approval was required.

## 2. Materials and methods

### 2.1. Data source and study design

Ethical approval and informed consent were not required, as this MR study used only publicly available, de-identified GWAS summary data, with original studies having obtained appropriate approvals.

The GWAS data used in this study were derived from published studies, and all study participants had already obtained informed consent in their respective original studies, so no additional ethical approval was required.

For the MR analyses, GWAS data for asthma were obtained from the UK Biobank database, containing 56,167 asthma cases and 3,52,255 controls. Asthma cases were diagnosed using self-reported questionnaires, hospital records (using ICD-9 and ICD-10 codes), and primary care records.^[13]^ GWAS data for CRS were obtained from the FinnGen database, a dataset that included 8524 patients with CRS and 1,67,849 controls. The diagnosis of CRS was determined by ICD-10 code J32. Both datasets are available in the IEU Open GWAS project (https://gwas.mrcieu.ac.uk/) under the GWAS IDs “ebi-a-GCST90014325” and “finn-b-J10_CHRONSINUSITIS,” respectively.

**Figure 1.**
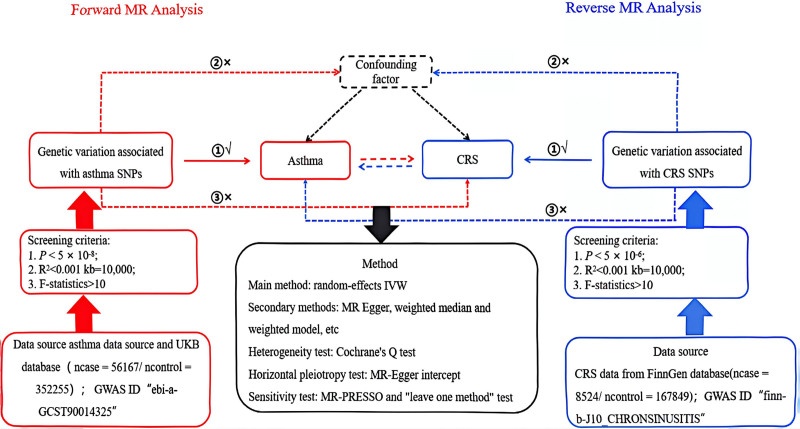
Flow chart of a 2-way 2-sample MR study of asthma and CRS. CRS = chronic rhinosinusitis, GWAS = genome-wide association study, IVW = inverse-variance weighted, MR = Mendelian randomization, SNPs = single nucleotide polymorphism.

To assess the causal relationship between asthma and CRS, a bidirectional 2-sample MR study was conducted using SNPs as an IVs. These SNPs must satisfy 3 assumptions: first, there is a strong correlation with exposure; second, the effect on outcome can only be achieved through exposure; third, there is no correlation with confounding factors.^[14]^ Figure 1 shows a flow diagram of the entire analysis process.

### 2.2. Selection of instrumental variables

In the forward MR analysis, a strict significance threshold (*P* < 5 × 10^−8^) was applied to indicate genome-wide level significance to select SNPs that were strongly associated with asthma.In the reverse MR, only 3 SNPs were found to be strongly associated with CRS at the significance threshold (*P* < 5 × 10^−8^). To include more SNPs, the significance threshold was set to (*P* < 5 × 10^−6^). To address issues caused by linkage disequilibrium, 2 key gene selection criteria were set: firstly, there must be a physical spacing of more than 10,000 base pairs between any 2 genes; and secondly, the level of linkage disequilibrium between these genes, as measured by the *r*^2^ value, must not exceed 0.001.^[15]^ To assess the likelihood of IV bias, the *F*-statistic was calculated, with an *F*-value exceeding 10 indicating the absence of such bias, and the *F*-statistic was calculated as *F* = β^2^/SE^2^.^[16]^ In addition, MR-PRESSO and Radial MR methods were used to detect possible outliers of SNPs, and the MR results were reassessed after removing these SNPs to ensure the reliability of the study^.[17,18]^

### 2.3. Statistical analysis

Two sample MR analyses were performed to investigate the causal relationship between asthma and chronic sinusitis using the “Two Sample MR” package in R software version 4.1.2. The primary MR analysis method used was the inverse-variance weighted (IVW) method,^[19]^ supplemented by complementary methods such as MR-Egger, weighted median and weighted mode for further analysis.^[20–22]^ When a *P* < .05 indicated an increased risk of outcome due to exposure, the causal effect of exposure and outcome was assessed using the ratio of odds ratios (OR). To assess heterogeneity, Cochran’s *Q* statistic was applied in both MR-Egger and IVW methods, and when the *P* > .05, heterogeneity was considered absent^.[23]^ We used the intercept *P* derived from the MR-Egger regression to check for horizontal pleiotropy, and a *P* > .05 indicated that there was no potential horizontal pleiotropy effect.^[20]^ In addition, sensitivity analyses were performed by the “leave-one-out” method and MR-PRESSO to demonstrate that individual SNPs do not overly influence the causal relationship between exposure and outcome.^[24,25]^

## 3. Results

### 3.1. The effect of asthma on CRS

**Figure 2.**
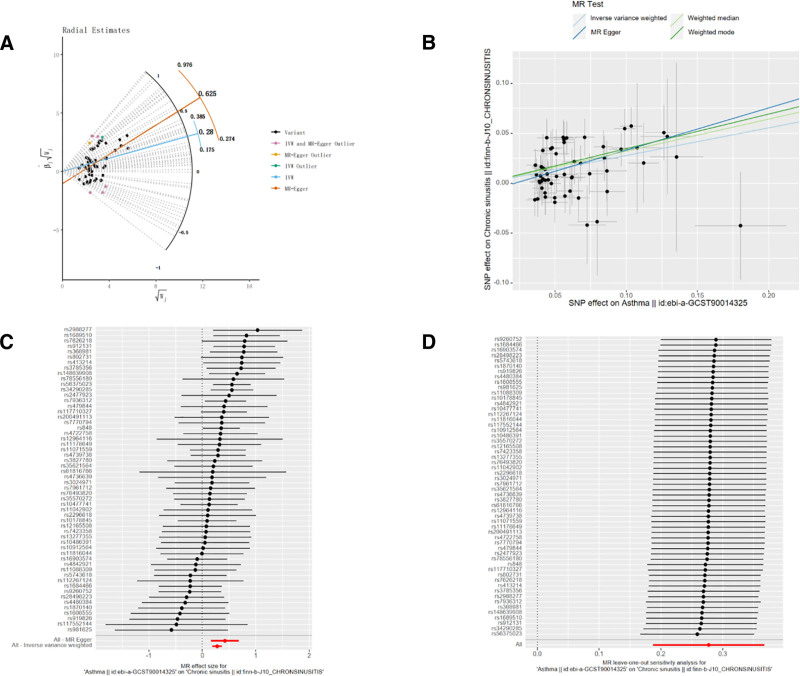
Forward MR analysis (A). Radial MR method (B) scatter plots of MR analysis for asthma and CRS, (C) forest maps of MR outcomes for asthma and CRS, (D) “leave-one-out” test for MR analysis of asthma and, CRS. CRS = chronic rhinosinusitis, MR = Mendelian randomization.

After rigorous screening and elimination of SNPs associated with chain disequilibrium, 7 outlier SNPs were detected using MR-PRESSO and Radial MR methods: rs992969, rs1444782, rs9272226, rs35441874, rs72823641, rs1837253, rs4795401. Finally 56 SNPs were found to be strongly associated with asthma, as shown in Figure 2A. All of these SNPs had *F* > 10, excluding bias from weak IVs. Information on relevant IVs can be found in Supplementary Material 1, Supplemental Digital Content, https://links.lww.com/MD/Q264. IVW results showed a positive correlation between asthma and CRS with an OR 95% confidence interval [CI] of 1.320 (95% CI = 1.206–1.444, *P* < .001), an OR 95% CI of 1.527 (95% CI = 1.178–1.978, *P* = .002) for the MR-Egger method, and a weighted median method result of 1.379 (95% CI = 1.204–1.578, *P* < .001), and the weighted mode method result was 1.414 (95% CI = 1.141–1.752, *P* = .002). These 3 complementary methods were consistent with the IVW results, which reinforces the positive correlation between asthma and CRS of chronic sinusitis, as shown in Figure 2B, C. Cochrane’s *Q* test revealed that the *Q* and *Q*_*P*_ values of IVW and MR-Egger were 57.720 (0.375) and 56.277 (0.390), respectively, with *Q*_*P*_ > .05, indicating that there was no heterogeneity in the MR results, as shown in Table 1.The value of Egger-intercept was −0.009, which was close to 0, with *P* = .244, indicating that there was no horizontal pleiotropy, as shown in Table 1. In addition, the results of the ‘leave-one-out’ method showed that after excluding individual SNPs one by one, there was no significant fluctuation in the IVW effect values of the remaining SNPs. This indicates that there are no SNPs in the IVs that strongly influence the results, further emphasizing the stability and confidence of the results, see Figure 2D.

**Table 1:** Results of heterogeneity and horizontal polytropy tests for forward and reverse MR.

Exposure	Outcome	Heterogeneity test		Horizontal pleiotropy test	MR-PRESSO	
		Cochran’s *Q* test	Rucker’s *Q* test	Egger intercept	Distortion test	Global test
		*P* value	*P* value	*P* value	Outliers	*P* value
		IVW	MR-Egger	MR-Egger		
Asthma	CRS	.375	.390	.244	60.055	.400
CRS	Asthma	.163	.137	.647	31.573	.167

CRS = chronic rhinosinusitis, MR = Mendelian randomization.

### 3.2. Effect of CRS on asthma

**Figure 3.**
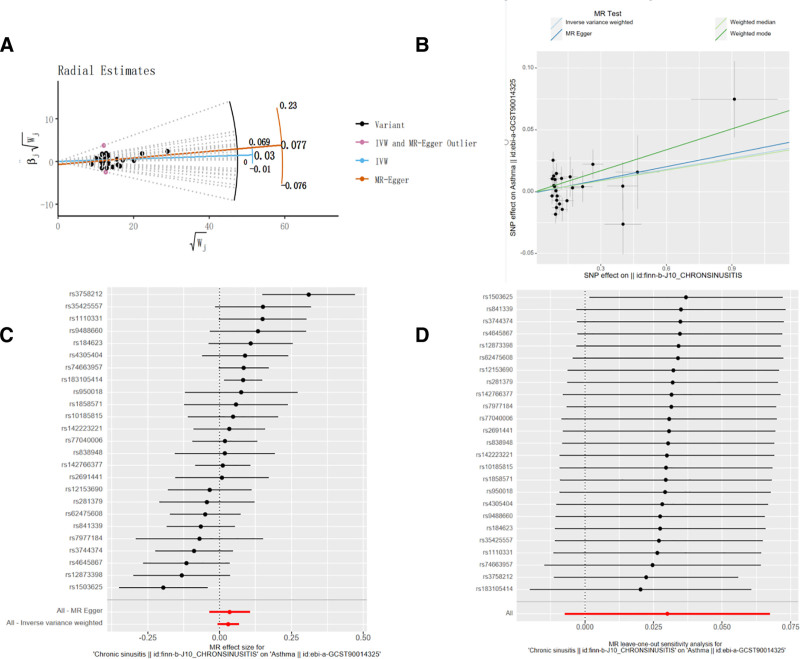
Reverse MR analysis (A). Radial MR method (B) scatter plots of MR analysis for asthma and CRS, (C) forest map of MR outcomes for asthma and CRS, (D) “leave-one-out” test for MR analysis of asthma and CRS. CRS = chronic rhinosinusitis, MR = Mendelian randomization.

The significance threshold is (*P* < 5 × 10^−6^), 2 outlier SNPs were detected using MR-PRESSO and Radial MR Methods: “rs1503625” and “rs3758212,” as shown in Figure 3A. Finally, 23 SNPs were found to be strongly associated IVs with CRS. These SNPs all had *F* > 10, excluding bias due to weak IVs. Information on the relevant IVs can be found in Supplementary Material 2, Supplemental Digital Content, https://links.lww.com/MD/Q264. The IVW results showed an OR with a 95% CI of 1.030 (95% CI = 0.998–1.062, *P* = .065), an MR-Egger method result with a 95% CI of 1.041 (95% CI = 0.983–1.103, *P* = .182), a weighted median method result of 1.030 (95% CI = 0.989–1.074, *P* = .156), and 1.063 (95% CI = 0.989–1.144, *P* = .111) for the weighted mode method result. The *P*-values for all 4 results were >.05, indicating that there was no positive correlation between CRS and asthma, as shown in Figure 3B, C. Cochrane’s *Q* test revealed that the *Q* and *Q*_*P*_ values of IVW and MR-Egger were 28.385 (0.163) and 28.097 (0.137), respectively, with *Q*_*P*_ > .05, indicating that there was no heterogeneity in the MR results, as shown in Table 1. The value of Egger-intercept was −0.001, which was close to 0, with *P = *.647, indicating that there was no horizontal pleiotropy, as shown in Table 1. In addition, the results of the “leave-one-out” method showed that after excluding individual SNPs one by one, there was no significant fluctuation in the IVW effect values of the remaining SNPs. This indicates that there are no SNPs in the IVs that strongly influence the results, further emphasizing the stability and confidence of the results, see Figure 3D.

## 4. Discussion

This study made full use of GWAS data on asthma and CRS and systematically assessed their genetic causality by 2-way 2-sample MR analysis. The findings showed a significant causal association between asthma and CRS with a OR of 1.340 (95% CI = 1.206–1.444, *P* < .001). However, no statistically significant causal association was observed for the relationship between CRS and asthma, with an OR of 1.041 (95% CI = 0.983–1.103, *P* = .182). This finding highlights the significant association between asthma and CRS at the genetic level, while excluding the effect of CRS on asthma.

The causal relationship between asthma and CRS has long been a hot topic of medical research. It has been reported that about 50% of patients with CRS are comorbid with asthma, whereas 40% to 75% of patients with asthma are comorbid with CRS^.[26]^ Although there is no direct evidence of a causal relationship between the 2 diseases, there appears to be a direct correlation between CRS severity and asthma severity. Sinus computed tomography (CT) scans have shown abnormal sinus mucosa in 84% of individuals with severe asthma, and a direct correlation has been found between sinus mucosal thickness and bronchial inflammation^.[27]^ However, it has been suggested that there is no correlation between CT-documented sinus involvement and asthma severity.^[28]^ In addition, Bresciani et al‘s CT results showed that CRS was not associated with asthma severity.^[29]^ These findings have not been widely recognized due to factors such as sample size, study design and population differences. In addition, there is no conclusive evidence that asthma directly causes CRS.

In terms of epidemiological causal inference, there are currently 3 main approaches: observational studies, randomized controlled trials (RCTs) and MR studies. Observational studies are susceptible to confounding factors and reverse causation, and therefore cannot readily assert causal effects of associations. Although RCTs are considered the gold standard for causal inference, their implementation is often constrained by limitations in medical ethics, trial design, and study costs. Conducting RCTs for potential risk factors is neither practical nor ethical in practice.^[30]^ In this context, MR studies can overcome the above research shortcomings and provide high-quality evidence for causal effects. Our MR results suggest that there is a significant association between asthma and CRS at the genetic level, whereas CRS has no effect on asthma.

The “one airway theory” proposes that the upper airways (e.g., sinuses) and lower airways (e.g., bronchi) are pathophysiologically continuous and may share similar inflammatory processes, suggesting that they may be driven by similar inflammatory mechanisms, particularly in terms of immune responses and cellular mediators.^[31]^ Thus, the link between the 2 diseases may not just manifest as a statistical association, but as a deep biological connection. In individuals with persistent chronic sinusitis, sinus tissue was examined pathologically. These results revealed several major tissue components similar to asthma, including inflammation of neutrophils and eosinophils, epithelial detachment, and basement membrane thickening.^[32]^ In addition, the shared pathophysiological mechanisms between asthma and CRS primarily involve T2-type inflammation, which is at the core of both airway diseases. T2-type inflammation is an inflammatory response driven by specific types of immune cells (e.g., eosinophils and basophils) and cytokines (e.g., interleukins 4, 5, 13). This inflammatory response plays a key role in the development of asthma and CRS, leading to airway hyperresponsiveness and tissue remodeling.^[33]^ These findings suggest that asthma may be associated with the development of CRS. Although it has also been suggested that increased levels of osteoprotegerin may make individuals with chronic sinusitis more susceptible to asthma.^[34]^ However, our MR findings did not find an increased risk of developing asthma with CRS at the genetic level. Further validation studies may be needed in the future.

Although a causal relationship between asthma and CRS was hypothesized by MR analysis in this study, there are some shortcomings in this study: as GWAS data mainly involves specific populations, its applicability in different races still needs further research; this study did not explore the specific biological mechanisms of asthma and CRS in depth, and experiments may be needed to validate it in the future; and this study did not find the causal relationship between CRS and asthma at the genetic level, and high-quality GWAS data or meta-analyses of GWAS may be needed to further clarify the relationship in the future.

## 5. Conclusion

Overall, the findings suggest a causal relationship between asthma and CRS, which may increase the risk of developing CRS. In clinical practice, clinicians should be more concerned about the possible occurrence of CRS in patients with asthma and take appropriate preventive measures.

## Acknowledgments

All genetic summary data were obtained from the IEU OpenGWAS project. We thank all participants and investigators for contributing to the GWAS data. We thank all the authors for their contributions in the study.

## Author contributions

**Conceptualization:** Jumei Li, Derong Zhu, Zhonglin Mu.

**Data curation:** Jumei Li, Zhonglin Mu.

**Formal analysis:** Jumei Li, Derong Zhu, Zhonglin Mu.

**Investigation:** Jumei Li, Zhonglin Mu.

**Methodology:** Jumei Li, Zhonglin Mu.

**Project administration:** Jumei Li, Zhonglin Mu.

**Resources:** Jumei Li, Derong Zhu, Zhonglin Mu.

**Software:** Jumei Li, Derong Zhu, Zhonglin Mu.

**Supervision:** Jumei Li, Zhonglin Mu.

**Validation:** Jumei Li, Zhonglin Mu.

**Visualization:** Jumei Li, Zhonglin Mu.

**Writing – original draft:** Jumei Li, Zhonglin Mu.

**Writing – review & editing:** Jumei Li, Zhonglin Mu.

## Supplementary Material


