# Supplementary material for: Causal relationship between asthma and chronic rhinosinusitis: A Mendelian randomization study
Source: Medicine (Baltimore). 2025 Oct 10;104(41):e45085. doi: 10.1097/MD.0000000000045085 (PMC12517928; doi:10.1097/MD.0000000000045085)
Supplement: Supplementary file 1 [file medi-104-e45085-s001.pdf]

SNP	effect_al	other_all	effect_al	other_all	beta.expos	beta.outc	eaf.expos	eaf.outco	id.outcom	chr	pos	se.outcom	pval.outcom	outcome	chr.expos	pos.exposu	se.exposure	pval.expos	samplesiz	id.exposu	exposure	R2	F	FMean	F_pow_bet
rs1017884	A	G	A	G	-0.061657	-0.0055	0.294552	0.3455	finn-b-J1	2	8443803	0.0173	0.7508	Chronic s	2	8443803	0.00717229	8.21864e-	408422	ebi-a-GCS	Asthma ||	0.0015798	646.26373578	498.85655	73.899833
rs1047774	G	T	G	T	0.074266	0.0095	0.128434	0.2077	finn-b-J1	5	131795310	0.0203	0.6402	Chronic s	5	131795310	0.00979669	3.43637e-	408422	ebi-a-GCS	Asthma ||	0.0012347	504.93321758	498.85655	57.467371
rs1048639	G	A	G	A	-0.038857	-0.0019	0.410809	0.4859	finn-b-J1	7	20376018	0.0164	0.9091	Chronic s	7	20376018	0.00665024	5.12743e-	408422	ebi-a-GCS	Asthma ||	0.0007309	298.7416964	498.85655	34.140557
rs1091256	T	C	T	C	0.0392578	6e-04	0.306507	0.3173	finn-b-J1	1	173170618	0.0176	0.9725	Chronic s	1	173170618	0.00711045	3.36814e-	408422	ebi-a-GCS	Asthma ||	0.0006551	267.7663686	498.85655	30.483003
rs1104290	T	C	T	C	0.0413305	0.0044	0.307493	0.3277	finn-b-J1	11	10655623	0.0175	0.8015	Chronic s	11	10655623	0.00711112	6.16936e-	408422	ebi-a-GCS	Asthma ||	0.0007274	297.3403025	498.85655	33.780440
rs1107155	T	C	T	C	-0.084687	-0.0249	0.128023	0.1546	finn-b-J1	15	61069988	0.0226	0.2717	Chronic s	15	61069988	0.00976462	4.21308e-	408422	ebi-a-GCS	Asthma ||	0.0016012	655.0336329	498.85655	75.218887
rs1108830	G	C	G	C	0.0604575	-0.0079	0.142544	0.1396	finn-b-J1	21	36464631	0.0237	0.7395	Chronic s	21	36464631	0.00931455	8.54673e-	408422	ebi-a-GCS	Asthma ||	0.0008934	365.2467527	498.85655	42.128559
rs1117864	T	G	T	G	-0.042926	-0.0138	0.407176	0.3585	finn-b-J1	12	71533238	0.0171	0.4206	Chronic s	12	71533238	0.00664703	1.06206e-	408422	ebi-a-GCS	Asthma ||	0.0008895	363.6320613	498.85655	41.703782
rs1122671	A	G	A	G	0.0428843	-0.0097	0.234223	0.1705	finn-b-J1	17	73828903	0.0218	0.6562	Chronic s	17	73828903	0.00769944	2.55035e-	408422	ebi-a-GCS	Asthma ||	0.0006597	269.6200045	498.85655	31.022612
rs1175521	T	C	T	C	0.079499	-0.0385	0.0667517	0.02561	finn-b-J1	19	3136091	0.0539	0.4752	Chronic s	19	3136091	0.0138067	8.51197e-	408422	ebi-a-GCS	Asthma ||	0.0007874	321.8562732	498.85655	33.154581
rs1177103	A	C	A	C	-0.126406	-0.0507	0.0669584	0.0989	finn-b-J1	19	33726578	0.0278	0.0679297	Chronic s	19	33726578	0.013395	3.84238e-	408422	ebi-a-GCS	Asthma ||	0.0019965	817.0457753	498.85655	89.053284
rs1181604	A	G	A	G	-0.047327	3e-04	0.325637	0.2559	finn-b-J1	10	6074082	0.0188	0.9874	Chronic s	10	6074082	0.00698072	1.20476e-	408422	ebi-a-GCS	Asthma ||	0.0009837	402.1667779	498.85655	45.963579
rs1216550	C	T	C	T	-0.045144	-0.0034	0.795068	0.7457	finn-b-J1	22	41819960	0.0188	0.8586	Chronic s	22	41819960	0.00809156	2.41674e-	408422	ebi-a-GCS	Asthma ||	0.0006641	271.4210288	498.85655	31.127153
rs1296411	G	A	G	A	0.107619	0.0354	0.0354621	0.01695	finn-b-J1	18	61442619	0.0648	0.585	Chronic s	18	61442619	0.0176522	1.08338e-	408422	ebi-a-GCS	Asthma ||	0.0007923	323.8492368	498.85655	37.168944
rs1327735	G	A	G	A	-0.04056	-0.0022	0.725959	0.6927	finn-b-J1	8	128777719	0.0178	0.9017	Chronic s	8	128777719	0.00732646	3.09286e-	408422	ebi-a-GCS	Asthma ||	0.0006545	267.5130015	498.85655	30.648380
rs1486399	AT	A	AT	A	-0.070735	-0.046	0.353557	0.2518	finn-b-J1	6	90963614	0.0189	0.0148898	Chronic s	6	90963614	0.00682025	3.35043e-	408422	ebi-a-GCS	Asthma ||	0.0022870	936.2350297	498.85655	107.56307
rs1608555	T	C	T	C	0.0377471	-0.0158	0.358532	0.314	finn-b-J1	7	22807473	0.0178	0.3722	Chronic s	7	22807473	0.00682176	3.14196e-	408422	ebi-a-GCS	Asthma ||	0.0006553	267.8501079	498.85655	30.617823
rs1684466	A	G	A	G	-0.056895	0.0129	0.637709	0.6113	finn-b-J1	3	196359310	0.0171	0.4522	Chronic s	3	196359310	0.00701248	4.9238e-1	408422	ebi-a-GCS	Asthma ||	0.0014957	611.8040043	498.85655	65.826668
rs1689510	C	G	C	G	0.0556484	0.046	0.340371	0.3187	finn-b-J1	12	56396768	0.0176	0.0089419	Chronic s	12	56396768	0.00688071	6.08555e-	408422	ebi-a-GCS	Asthma ||	0.0013905	568.7206295	498.85655	65.409202
rs1690357	G	C	G	C	0.0865509	-0.0083	0.0765121	0.1271	finn-b-J1	5	14610309	0.0248	0.7388	Chronic s	5	14610309	0.0125488	5.30518e-	408422	ebi-a-GCS	Asthma ||	0.0010586	432.8141942	498.85655	47.570616
rs1870140	G	A	G	A	-0.049794	0.0191	0.845758	0.8027	finn-b-J1	10	82246749	0.0207	0.3561	Chronic s	10	82246749	0.00906558	3.95977e-	408422	ebi-a-GCS	Asthma ||	0.0006468	264.3752662	498.85655	30.169134
rs2004911	GTAGA	G	GTAGA	G	-0.128778	-0.0468	0.032041	0.0207	finn-b-J1	9	101932851	0.058	0.4193	Chronic s	9	101932851	0.0187364	6.28058e-	408422	ebi-a-GCS	Asthma ||	0.0010286	420.5623092	498.85655	47.240126
rs2296618	G	A	G	A	-0.06188	-0.0062	0.135293	0.09165	finn-b-J1	1	198666232	0.0286	0.8291	Chronic s	1	198666232	0.0096286	1.30434e-	408422	ebi-a-GCS	Asthma ||	0.0008959	366.2421890	498.85655	41.302040
rs2477923	C	T	C	T	-0.036131	-0.0181	0.463296	0.4924	finn-b-J1	10	8565990	0.0164	0.2697	Chronic s	10	8565990	0.00656022	3.63672e-	408422	ebi-a-GCS	Asthma ||	0.0006492	265.3258707	498.85655	30.334047
rs2849822	T	C	T	C	0.0497316	-0.0146	0.280804	0.3038	finn-b-J1	14	68728425	0.0179	0.4151	Chronic s	14	68728425	0.00731458	1.05366e-	408422	ebi-a-GCS	Asthma ||	0.0009989	408.4006932	498.85655	46.225974
rs2988277	T	C	T	C	-0.044119	-0.0456	0.398654	0.2596	finn-b-J1	1	167431352	0.0188	0.0154099	Chronic s	1	167431352	0.00666025	3.49221e-	408422	ebi-a-GCS	Asthma ||	0.0009332	381.5104913	498.85655	43.879572
rs3024971	G	T	G	T	-0.112196	-0.0203	0.107142	0.04439	finn-b-J1	12	57493727	0.04	0.611999	Chronic s	12	57493727	0.0106267	4.67089e-	408422	ebi-a-GCS	Asthma ||	0.0024083	986.0095008	498.85655	111.46997
rs3429028	A	G	A	G	-0.098872	-0.0547	0.255414	0.2167	finn-b-J1	2	242698640	0.0201	0.00638793	Chronic s	2	242698640	0.00750984	1.3842e-3	408422	ebi-a-GCS	Asthma ||	0.0037181	1524.254510	498.85655	173.33320
rs3557027	T	G	T	G	0.0509447	0.0068	0.396277	0.3555	finn-b-J1	3	33047662	0.0172	0.6924	Chronic s	3	33047662	0.00670108	2.90536e-	408422	ebi-a-GCS	Asthma ||	0.0012418	507.8217070	498.85655	57.797414
rs3562156	G	A	G	A	-0.044391	-0.0092	0.365249	0.452	finn-b-J1	7	20586843	0.0166	0.577301	Chronic s	7	20586843	0.00681856	7.49894e-	408422	ebi-a-GCS	Asthma ||	0.0009137	373.5204023	498.85655	42.384061
rs368981	A	G	A	G	0.0561843	0.0436	0.708867	0.709	finn-b-J1	9	6139009	0.0181	0.016	Chronic s	9	6139009	0.0072267	7.57182e-	408422	ebi-a-GCS	Asthma ||	0.0013029	532.8308451	498.85655	60.443540
rs3785356	T	C	T	C	0.0563	0.0409	0.29795	0.2618	finn-b-J1	16	27349168	0.0186	0.02776	Chronic s	16	27349168	0.00716317	3.85212e-	408422	ebi-a-GCS	Asthma ||	0.0013260	542.3023619	498.85655	61.774078
rs3827780	A	G	A	G	-0.036847	-0.0086	0.555099	0.5837	finn-b-J1	6	135709760	0.0167	0.608001	Chronic s	6	135709760	0.00656749	2.01683e-	408422	ebi-a-GCS	Asthma ||	0.0006706	274.0775777	498.85655	31.478389
rs413214	A	G	A	G	0.0469332	0.0346	0.616158	0.6564	finn-b-J1	5	141530157	0.0173	0.0452199	Chronic s	5	141530157	0.00671099	2.68164e-	408422	ebi-a-GCS	Asthma ||	0.0010419	425.9853375	498.85655	48.908815
rs4480384	G	A	G	A	0.043097	-0.0138	0.643257	0.7007	finn-b-J1	1	8659714	0.0179	0.4413	Chronic s	1	8659714	0.00681273	2.51675e-	408422	ebi-a-GCS	Asthma ||	0.0008524	348.4507674	498.85655	40.017663
rs4722758	G	C	G	C	0.0634119	0.0216	0.19958	0.1564	finn-b-J1	7	28156606	0.0226	0.3378	Chronic s	7	28156606	0.00814979	7.20775e-	408422	ebi-a-GCS	Asthma ||	0.0012847	525.3778776	498.85655	60.540875
rs4736639	C	G	C	G	-0.039726	-0.0072	0.286228	0.2044	finn-b-J1	8	134185916	0.0205	0.7249	Chronic s	8	134185916	0.0072377	4.04809e-	408422	ebi-a-GCS	Asthma ||	0.0006448	263.5324266	498.85655	30.126185
rs4739738	A	G	A	G	-0.067912	-0.0199	0.641572	0.6929	finn-b-J1	8	81291645	0.0178	0.2639	Chronic s	8	81291645	0.00680464	1.85994e-	408422	ebi-a-GCS	Asthma ||	0.0021211	868.1595531	498.85655	99.605365
rs479844	G	A	G	A	0.0403223	0.0164	0.554743	0.5905	finn-b-J1	11	65551957	0.0167	0.3269	Chronic s	11	65551957	0.00655551	7.70407e-	408422	ebi-a-GCS	Asthma ||	0.0008031	328.3062430	498.85655	37.833596
rs4842921	A	G	A	G	-0.040709	0.0049	0.387483	0.3332	finn-b-J1	15	84556623	0.0174	0.780301	Chronic s	15	84556623	0.00669088	1.17001e-	408422	ebi-a-GCS	Asthma ||	0.0007866	321.5413595	498.85655	37.018667
rs5637502	A	G	A	G	0.103455	0.0573	0.237326	0.264	finn-b-J1	15	67448363	0.0186	0.00204099	Chronic s	15	67448363	0.00769319	3.1798e-4	408422	ebi-a-GCS	Asthma ||	0.0038745	1588.583696	498.85655	180.83814
rs5743618	A	C	A	C	-0.066445	0.0148	0.225707	0.1535	finn-b-J1	4	38798648	0.023	0.5201	Chronic s	4	38798648	0.00786149	2.86484e-	408422	ebi-a-GCS	Asthma ||	0.0015431	631.2189744	498.85655	71.435199
rs6181676	C	T	C	T	0.135284	0.0263	0.033244	0.007875	finn-b-J1	1	152319572	0.0948	0.7815	Chronic s	1	152319572	0.0186871	4.50609e-	408422	ebi-a-GCS	Asthma ||	0.0011763	481.0289564	498.85655	52.409383
rs7423358	C	T	C	T	0.0430394	0.003	0.758113	0.7142	finn-b-J1	2	228704721	0.0182	0.8692	Chronic s	2	228704721	0.00764835	1.83071e-	408422	ebi-a-GCS	Asthma ||	0.0006793	277.6585389	498.85655	31.666269
rs7626218	T	A	T	A	-0.041353	-0.0328	0.395198	0.3785	finn-b-J1	3	176852038	0.0169	0.0526405	Chronic s	3	176852038	0.00667906	5.95937e-	408422	ebi-a-GCS	Asthma ||	0.0008174	334.1508519	498.85655	38.334672
rs7649382	G	C	G	C	0.0863742	0.0123	0.0579568	0.08036	finn-b-J1	5	156691025	0.0304	0.684699	Chronic s	5	156691025	0.0140655	8.20881e-	408422	ebi-a-GCS	Asthma ||	0.0008146	332.9923329	498.85655	37.710103
rs7770794	A	G	A	G	0.0415551	0.0151	0.285802	0.3479	finn-b-J1	6	154627823	0.0172	0.3819	Chronic s	6	154627823	0.0072243	8.81211e-	408422	ebi-a-GCS	Asthma ||	0.0007049	288.1216016	498.85655	33.086979
rs7855618	T	G	T	G	0.0506806	0.0295	0.146826	0.1296	finn-b-J1	1	173057239	0.0246	0.2311	Chronic s	1	173057239	0.00922962	3.99485e-	408422	ebi-a-GCS	Asthma ||	0.0006435	262.9908469	498.85655	30.151981
rs7936312	T	G	T	G	0.0838325	0.0366	0.476762	0.4144	finn-b-J1	11	76293726	0.0166	0.0277	Chronic s	11	76293726	0.00652802	9.54333e-	408422	ebi-a-GCS	Asthma ||	0.0035063	1437.104032	498.85655	164.91565
rs7961712	A	G	A	G	0.0564852	0.0088	0.849901	0.8639	finn-b-J1	12	94604963	0.0239	0.7136	Chronic s	12	94604963	0.00917211	7.34971e-	408422	ebi-a-GCS	Asthma ||	0.0008140	332.7408749	498.85655	37.925457
rs802731	G	C	G	C	0.0479182	0.0355	0.270854	0.2554	finn-b-J1	6	128279429	0.0188	0.0589006	Chronic s	6	128279429	0.00733727	6.54184e-	408422	ebi-a-GCS	Asthma ||	0.0009069	370.7505461	498.85655	42.651272
rs848	C	A	C	A	-0.096334	-0.034	0.817872	0.6315	finn-b-J1	5	131996500	0.017	0.0460098	Chronic s	5	131996500	0.00847156	5.79696e-	408422	ebi-a-GCS	Asthma ||	0.0027647	1132.307085	498.85655	129.31116
rs912131	G	A	G	A	0.0580773	0.0454	0.703887	0.6411	finn-b-J1	13	100032346	0.0171	0.00790806	Chronic s	13	100032346	0.00713704	4.03738e-	408422	ebi-a-GCS	Asthma ||	0.0014060	575.0708103	498.85655	66.218078
rs919826	C	T	C	T	-0.035753	0.0166	0.489954	0.502	finn-b-J1	9	23586654	0.0164	0.313	Chronic s	9	23586654	0.00654304	4.649e-08	408422	ebi-a-GCS	Asthma ||	0.0006388	261.0939966	498.85655	29.857850
rs9260752	A	G	A	G	0.180227	-0.0425	0.0211877	0.02389	finn-b-J1	6	29935010	0.0539	0.4305	Chronic s	6	29935010	0.0320378	1.85042e-	408422	ebi-a-GCS	Asthma ||	0.0013472	550.9921878	498.85655	31.645672
rs981625	G	C	G	C	0.0724725	-0.0419	0.06495	0.04665	finn-b-J1	13	74039935	0.0392	0.2846	Chronic s	13	74039935	0.0132157	4.16188e-	408422	ebi-a-GCS	Asthma ||	0.0006379	260.7201645	498.85655	30.072265

SNP	effect_al	other_alle	effect_al	other_alle	beta.expos	beta.outc	eaf.expos	eaf.outco	remove	palindrom	ambiguous	id.outcome
rs1018581	A	G	A	G	-0.0887	-0.004176	0.2466	0.303093	FALSE	FALSE	FALSE	ebi-a-GCS
rs1110331	A	G	A	G	-0.0849	-0.012724	0.6517	0.574549	FALSE	FALSE	FALSE	ebi-a-GCS
rs1215369	A	T	A	T	-0.1008	0.0034682	0.2029	0.252971	FALSE	TRUE	FALSE	ebi-a-GCS
rs1287339	T	C	T	C	-0.0977	0.012846	0.19	0.186185	FALSE	FALSE	FALSE	ebi-a-GCS
rs1422232	T	C	T	C	0.4682	0.0158373	0.007318	0.0126948	FALSE	FALSE	FALSE	ebi-a-GCS
rs1427663	A	C	A	C	0.401	0.0044879	0.0148	0.0300789	FALSE	FALSE	FALSE	ebi-a-GCS
rs1831054	A	T	A	T	0.9119	0.0748749	0.00217	0.0121294	FALSE	TRUE	FALSE	ebi-a-GCS
rs184623	G	A	G	A	-0.091	-0.00986	0.3466	0.363699	FALSE	FALSE	FALSE	ebi-a-GCS
rs1858571	A	G	A	G	0.0856	0.0048974	0.2688	0.224791	FALSE	FALSE	FALSE	ebi-a-GCS
rs2691441	T	C	T	C	0.0953	0.0007827	0.2293	0.216679	FALSE	FALSE	FALSE	ebi-a-GCS
rs281379	A	G	A	G	0.0774	-0.003416	0.4083	0.52429	FALSE	FALSE	FALSE	ebi-a-GCS
rs3542555	A	G	A	G	-0.0974	-0.014717	0.1899	0.189005	FALSE	FALSE	FALSE	ebi-a-GCS
rs3744374	A	G	A	G	-0.1116	0.0099161	0.2307	0.235143	FALSE	FALSE	FALSE	ebi-a-GCS
rs4305404	A	G	A	G	0.1208	0.0107163	0.1212	0.144363	FALSE	FALSE	FALSE	ebi-a-GCS
rs4645867	C	T	C	T	0.124	-0.014308	0.1084	0.149422	FALSE	FALSE	FALSE	ebi-a-GCS
rs6247560	C	T	C	T	0.1456	-0.007292	0.07437	0.151499	FALSE	FALSE	FALSE	ebi-a-GCS
rs7466395	C	T	C	T	0.2642	0.022158	0.03316	0.0834186	FALSE	FALSE	FALSE	ebi-a-GCS
rs7704000	A	C	A	C	0.2172	0.004044	0.03465	0.0760935	FALSE	FALSE	FALSE	ebi-a-GCS
rs7977184	G	A	G	A	-0.0988	0.0069126	0.1778	0.0943967	FALSE	FALSE	FALSE	ebi-a-GCS
rs838948	A	G	A	G	0.1709	0.0031116	0.052	0.0491606	FALSE	FALSE	FALSE	ebi-a-GCS
rs841339	A	G	A	G	-0.4021	0.0262484	0.99	0.981566	FALSE	FALSE	FALSE	ebi-a-GCS
rs9488660	G	A	G	A	-0.0782	-0.010462	0.4357	0.395155	FALSE	FALSE	FALSE	ebi-a-GCS
rs950018	G	A	G	A	0.1601	0.0120469	0.06066	0.0475535	FALSE	FALSE	FALSE	ebi-a-GCS

SNP	chr	pos	se.outcome	samplesize	pval.outc	outcome	originalna	outcome.de	mr_keep.ou	data_sourc	samplesize	pval.expos
rs1018581	2	42973439	0.0071145	408422	0.557256	Asthma ||	Asthma	Asthma ||	TRUE	igd	176373	3.72e-06
rs1110331	16	89774396	0.0066368	408422	0.0552128	Asthma ||	Asthma	Asthma ||	TRUE	igd	176373	1.03e-06
rs1215369	5	117273679	0.0075144	408422	0.644405	Asthma ||	Asthma	Asthma ||	TRUE	igd	176373	9.96e-07
rs1287339	13	93032056	0.0083961	408422	0.12602	Asthma ||	Asthma	Asthma ||	TRUE	igd	176373	3.33e-06
rs1422232	2	136958333	0.029834	408422	0.595524	Asthma ||	Asthma	Asthma ||	TRUE	igd	176373	3.52e-06
rs1427663	19	14062446	0.0198369	408422	0.821016	Asthma ||	Asthma	Asthma ||	TRUE	igd	176373	1.59e-08
rs1831054	7	122472937	0.030918	408422	0.0154469	Asthma ||	Asthma	Asthma ||	TRUE	igd	176373	4.36e-06
rs184623	19	41703955	0.0067877	408422	0.146313	Asthma ||	Asthma	Asthma ||	TRUE	igd	176373	1.34e-07
rs1858571	10	29634588	0.0079156	408422	0.536109	Asthma ||	Asthma	Asthma ||	TRUE	igd	176373	3.77e-06
rs2691441	1	58002491	0.0079094	408422	0.921176	Asthma ||	Asthma	Asthma ||	TRUE	igd	176373	1.04e-06
rs281379	19	49214274	0.0065518	408422	0.60212	Asthma ||	Asthma	Asthma ||	TRUE	igd	176373	3.41e-06
rs3542555	1	77874558	0.0083216	408422	0.0769644	Asthma ||	Asthma	Asthma ||	TRUE	igd	176373	3.59e-06
rs3744374	17	34072555	0.0076861	408422	0.197008	Asthma ||	Asthma	Asthma ||	TRUE	igd	176373	1.09e-08
rs4305404	3	15577567	0.0092536	408422	0.246835	Asthma ||	Asthma	Asthma ||	TRUE	igd	176373	1.51e-06
rs4645867	14	75749794	0.0095746	408422	0.135074	Asthma ||	Asthma	Asthma ||	TRUE	igd	176373	4.26e-06
rs6247560	7	158632467	0.0091458	408422	0.425306	Asthma ||	Asthma	Asthma ||	TRUE	igd	176373	4.33e-06
rs7466395	3	193988454	0.0117917	408422	0.0602282	Asthma ||	Asthma	Asthma ||	TRUE	igd	176373	2.19e-08
rs7704000	2	78199385	0.0125971	408422	0.748192	Asthma ||	Asthma	Asthma ||	TRUE	igd	176373	2.09e-06
rs7977184	12	63312948	0.011167	408422	0.535903	Asthma ||	Asthma	Asthma ||	TRUE	igd	176373	4.49e-06
rs838948	12	125178410	0.015189	408422	0.83768	Asthma ||	Asthma	Asthma ||	TRUE	igd	176373	3.36e-06
rs841339	1	95142843	0.0244156	408422	0.282345	Asthma ||	Asthma	Asthma ||	TRUE	igd	176373	2.06e-06
rs9488660	6	97823737	0.0066883	408422	0.117772	Asthma ||	Asthma	Asthma ||	TRUE	igd	176373	2.4e-06
rs950018	12	87860883	0.0160359	408422	0.452504	Asthma ||	Asthma	Asthma ||	TRUE	igd	176373	3.9e-06

SNP	se.exposu	pos.expos	chr.expos	id.exposu	exposure	mr_keep.e	pval_orig	data_sourc	action	mr_keep	R2	F	FMean	F_pow_beta_se
rs1018581	0.0192	42973439	2	finn-b-J1	|| id:fin	TRUE	reported	igd	2	TRUE	0.0029234	517.12388	580.64575	21.3424750434028
rs1110331	0.0174	89774396	16	finn-b-J1	|| id:fin	TRUE	reported	igd	2	TRUE	0.0032722	579.02484	580.64575	23.8076694411415
rs1215369	0.0206	117273679	5	finn-b-J1	|| id:fin	TRUE	reported	igd	2	TRUE	0.0032865	581.57106	580.64575	23.943444245452
rs1287339	0.021	93032056	13	finn-b-J1	|| id:fin	TRUE	reported	igd	2	TRUE	0.002938	519.71203	580.64575	21.6446485260771
rs1422232	0.101	136958333	2	finn-b-J1	|| id:fin	TRUE	reported	igd	2	TRUE	0.0031848	563.51818	580.64575	21.4891912557592
rs1427663	0.071	14062446	19	finn-b-J1	|| id:fin	TRUE	reported	igd	2	TRUE	0.0046892	830.94704	580.64575	31.8986312239635
rs1831054	0.1985	122472937	7	finn-b-J1	|| id:fin	TRUE	reported	igd	2	TRUE	0.0036011	637.43319	580.64575	21.1044194176728
rs184623	0.0172	41703955	19	finn-b-J1	|| id:fin	TRUE	reported	igd	2	TRUE	0.0037507	664.01768	580.64575	27.9914818820984
rs1858571	0.0185	29634588	10	finn-b-J1	|| id:fin	TRUE	reported	igd	2	TRUE	0.0028803	509.47498	580.64575	21.4093791088386
rs2691441	0.0195	58002491	1	finn-b-J1	|| id:fin	TRUE	reported	igd	2	TRUE	0.0032100	567.97434	580.64575	23.884523339908
rs281379	0.0167	49214274	19	finn-b-J1	|| id:fin	TRUE	reported	igd	2	TRUE	0.0028946	512.01064	580.64575	21.4807271684177
rs3542555	0.021	77874558	1	finn-b-J1	|| id:fin	TRUE	reported	igd	2	TRUE	0.0029188	516.30719	580.64575	21.5119274376417
rs3744374	0.0195	34072555	17	finn-b-J1	|| id:fin	TRUE	reported	igd	2	TRUE	0.0044208	783.16465	580.64575	32.7536094674556
rs4305404	0.0251	15577567	3	finn-b-J1	|| id:fin	TRUE	reported	igd	2	TRUE	0.0031085	549.96599	580.64575	23.1625529753496
rs4645867	0.027	75749794	14	finn-b-J1	|| id:fin	TRUE	reported	igd	2	TRUE	0.0029721	525.76612	580.64575	21.0919067215364
rs6247560	0.0317	158632467	7	finn-b-J1	|| id:fin	TRUE	reported	igd	2	TRUE	0.0029186	516.27910	580.64575	21.0961995840341
rs7466395	0.0472	193988454	3	finn-b-J1	|| id:fin	TRUE	reported	igd	2	TRUE	0.0044757	792.93955	580.64575	31.3315318873887
rs7704000	0.0458	78199385	2	finn-b-J1	|| id:fin	TRUE	reported	igd	2	TRUE	0.0031560	558.39003	580.64575	22.4899601456875
rs7977184	0.0215	63312948	12	finn-b-J1	|| id:fin	TRUE	reported	igd	2	TRUE	0.0028539	504.80254	580.64575	21.1172309356409
rs838948	0.0368	125178410	12	finn-b-J1	|| id:fin	TRUE	reported	igd	2	TRUE	0.0028795	509.33715	580.64575	21.5669379135161
rs841339	0.0847	95142843	1	finn-b-J1	|| id:fin	TRUE	reported	igd	2	TRUE	0.0032014	566.43890	580.64575	22.5372709291353
rs9488660	0.0166	97823737	6	finn-b-J1	|| id:fin	TRUE	reported	igd	2	TRUE	0.0030070	531.95660	580.64575	22.1920452895921
rs950018	0.0347	87860883	12	finn-b-J1	|| id:fin	TRUE	reported	igd	2	TRUE	0.0029210	516.69648	580.64575	21.2874535956615
